# Treatment of consistent BRAF/HRAS gene mutation and MYC amplification radiation-induced abdominal wall angiosarcoma with low-dose apatinib: a case report

**DOI:** 10.1186/s12885-019-6351-4

**Published:** 2019-12-05

**Authors:** Jieshan Guan, Zhijie Luo, Zhiwei Xiao, Yubin Xie, Lizhu Lin

**Affiliations:** 1 Cancer Center, The First Affiliated Hospital of Guangzhou University of Chinese Medicine, 510405 Guangzhou, Guangdong People’s Republic of China; 2 The First Clinical Medical College, Guangzhou University of Chinese Medicine, 510405 Guangzhou, Guangdong People’s Republic of China

**Keywords:** Radiation-induced angiosarcoma, Secondary angiosarcoma, Apatinib, Tyrosine kinase inhibitor

## Abstract

**Background:**

An extremely rare condition, radiation-induced angiosarcoma is characterized by a poor prognosis, high recurrence rate and lack of effective treatment. Herein, we present a case report of a 48-year-old female patient with radiation-induced abdominal wall angiosarcoma who showed a dramatic response to low-dose apatinib.

**Case presentation:**

The patient, who was diagnosed with cervical squamous cell carcinoma 20 years ago, had received radiotherapy and chemotherapy after operation. Angiosarcomas of the abdominal wall appeared 9 years later. After repeated surgical operations and intravenous chemotherapy for the angiosarcomas, the patient developed tumor recurrence and pulmonary metastasis. The abdominal wall tumors showed repeated rupture and bleeding, with poor wound healing. On evaluation, laboratory findings detected the negative serum tumor markers CEA, CA 125, CA 15–3 and CA 19–9. Imaging showed multiple subcutaneous nodules and masses in the abdominal wall, accompanied by suspected small subpleural nodule at the lower lobe of the right lung. Immunohistochemistry of previous surgical pathology indicated that CD31, ERG and Vim were positive. The result of whole exome sequencing suggested the mutations of BRAF and HRAS, and the amplification of MYC. Based on the above results, the patient was clinically diagnosed with radiation-induced angiosarcoma of the abdominal wall with pulmonary metastasis. The patient was treated with low-dose apatinib and rejected reoperation or chemotherapy.

**Results:**

At the 6-month follow-up visit, the abdominal wall lesions that had previously ruptured stopped bleeding and showed significant shrinkage. Imaging showed that most of the abdominal wall lesions had partially regressed, and some of the lesions on the abdominal wall and the suspected lesion of subpleural nodule at the lower lobe of the right lung had disappeared.

**Conclusions:**

We described this case and reviewed the literature on radiation-related angiosarcoma. Importantly, this case suggests that apatinib may be an effective and sensitive treatment for radiation-induced angiosarcoma even at the lowest dosage, without aggravating the bleeding of lesions.

## Background

Angiosarcoma (AS) is a rare malignant tumor of vascular endothelial cell differentiation, which mostly affects female patients. The 5-year survival rate of AS is less than 30% [1], and it is prone to recurrence after surgery. There is still a lack of standard treatments for AS; the current therapies mainly include surgical resection, radiotherapy, chemotherapy and targeted therapy. Secondary AS may be associated with radiotherapy or chronic lymphedema, which may be distinguished from primary tumors by MYC gene amplification [2, 3]. Abdominal wall AS associated with radiotherapy was first reported by Richard Komorowski in 1976 [4], and it remains so rare that there is no mutation data or effective therapies.

Apatinib (Hengrui Pharmaceutical Co., Ltd., Shanghai, China) is a small tyrosine kinase inhibitor that targets vascular endothelial growth factor receptor 2 (VEGF-R2) [5]. Here, we report the case of a 48-year-old female patient with radiation-induced abdominal wall AS who showed an excellent response to low-dose apatinib.

## Case representation

The patient had a history of HPV-42 infection and cervical cancer. There was no family history of cancer. At the age of 38, she underwent a hysterectomy, and the postoperative pathology revealed large cell non-keratinizing squamous cell carcinoma of the cervix, which was in Stage IIA. Concurrent chemo-radiotherapy was performed postoperatively (total pelvic radiotherapy, DT 5040 cGy/28 times, concurrent 2 courses of cisplatin chemotherapy), followed by 4 courses of doublet chemotherapy (paclitaxel 240 mg day 1 + cisplatin 30 mg day 1–4). No evidence of tumor recurrence or the metastasis of cervical cancer was found in the follow-up examinations.

The patient was admitted to a local hospital in Shenzhen for treatment of an abdominal mass rupture. Core biopsy suggested the possibility of a low-differentiated tumor. Immunohistochemistry (IHC): P16(+), Ki-67 (almost 40%+), CK, CAM5.2, P40, CK8/18, ER and PR were all negative. The patient underwent abdominal wall tumor resection 2 times after admission for 1 month. Both operations performed in the lower abdominal wall. Due to positive margin of the first operation, the patient underwent the second operation to expand the scope. The result of frozen pathological sections during the second operation indicated free surgical margin showed free surgical margin of the low-differentiated AS. IHC: CD31, ERG and Vim were positive, while S-100, CD56, SOX10, HMB45, MelanA, EMA, CK5/6 and CD34 were all negative. She received 2 courses of doublet chemotherapy (docetaxel 60 mg day 1 and 8 + gemcitabine 1.2 g day 1 and 8) 2 months after the later operation.

Due to poor wound healing after surgery, the patient underwent another two operations for repair of chronic ulcer of lower abdominal wall and reconstruction of skin flap adjacent to abdominal wall 3 months and four months after the later operation. The wound was infected and the local skin flap showed ischemic necrosis within a limited range. The pathological findings were consistent with the previous diagnosis of AS. Multiple abdominal wall masses appeared in 2 months after the reconstruction of skin flap adjacent to abdominal wall, gradually increasing, and rupture and bleeding occurred in next month.

**Fig. 1:**
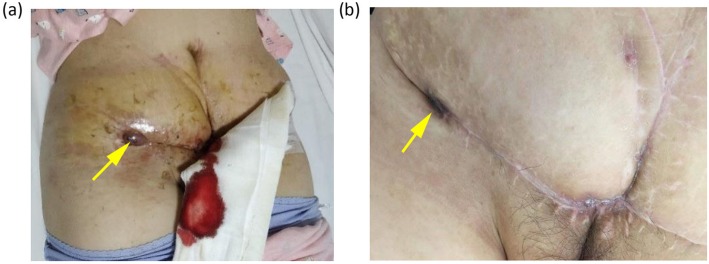
**a** Rupture and bleeding of the abdominal wall mass before anti-VEGF treatment. **b** The tumor stopped bleeding and significantly shrank 2 months after apatinib

One month after the occurrence of rupture and bleeding, the patient complained of severe pain from the recurrent ulcerated lesions of the lower abdominal wall. A physical examination revealed a 2 × 3 cm mass in the abdominal wall of the right lower abdomen, and a 1 × 1 cm purple mass in the middle and lower abdomen accompanied by superficial ulcer and bleeding (Fig. 1a). There was a soft-tissue mass of about 1 × 1 cm in size at the left iliac fossa and inguinal region. On evaluation, laboratory findings detected negative serum tumor markers of CEA, CA 125, CA 15–3 and CA 19–9. The result of whole exome sequencing (WES) carried out by the 3D Medicines Corporation (China) indicated mutations of BRAF p. G469 V (mutation abundance: 45.30%), HRAS E62_S65dup (mutation abundance: 19.80%), and the amplification of MYC (copy number alteration: 4-fold change), accompanied by a low tumor mutation burden (TMB: 1.77 Muts/Mb) and microsatellite stability (MSS) status. The results also indicated that no mutations or alternations in the copy number of VEGFA, VEGF-R2 (KDR), VEGF-R1 (FLT1), VEGF-R3 (FLT4), TIE or TEK (TIE2) were detected in the tissue sample.

**Fig. 2:**
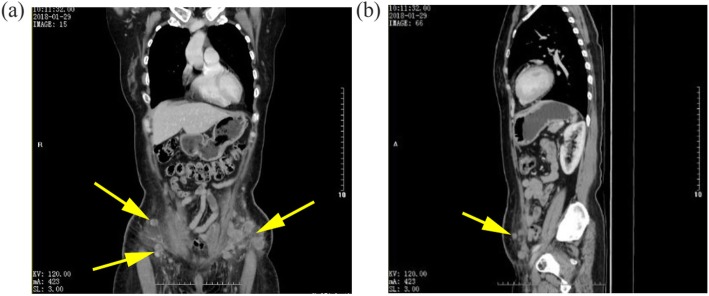
Contrasted CT scans showing multiple nodules and masses in the lower abdominal wall. **a** Coronal plane. **b** Sagittal plane

The thoracic and abdominal contrast-enhanced computed tomography (CT) scans indicated multiple subcutaneous masses on the abdominal wall (Fig. 2) and a suspected small subpleural nodule at the lower lobe of the right lung. Due to the rupture and bleeding of the masses, the patient began to take low-dose apatinib treatment (250 mg orally once daily), in combination with hemostasis, wound dressing and traditional Chinese medicine treatment. The purpose of traditional Chinese medicine treatment was to help the patient recover from the poor status after previous operations and chemotherapies.

**Fig. 3:**
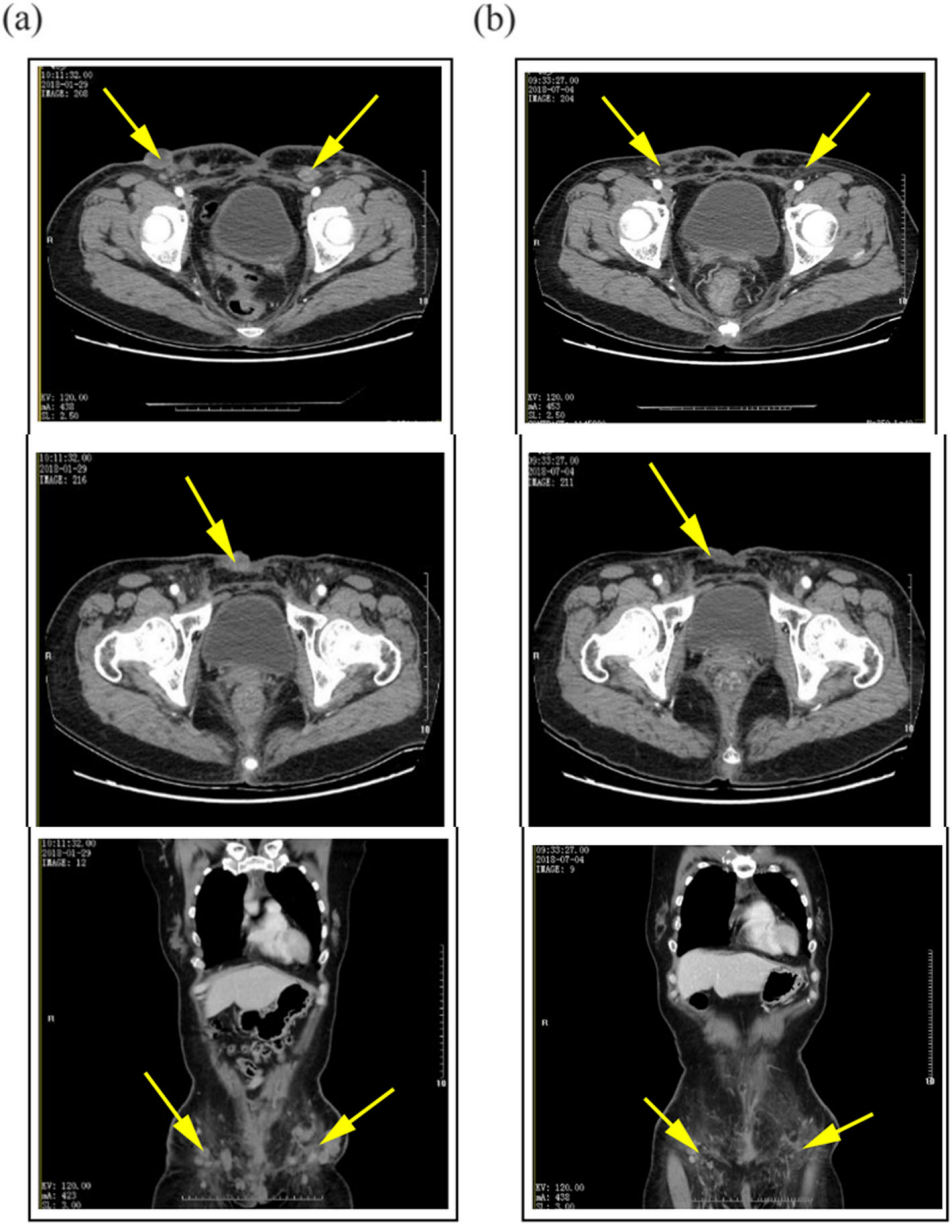
Contrasted CT scans showing the size and number of abdominal wall tumors decreased significantly after 6 months of oral apatinib treatment (axial and coronal). **a** Before treatment. **b** After 6 months of apatinib

**Fig. 4:**
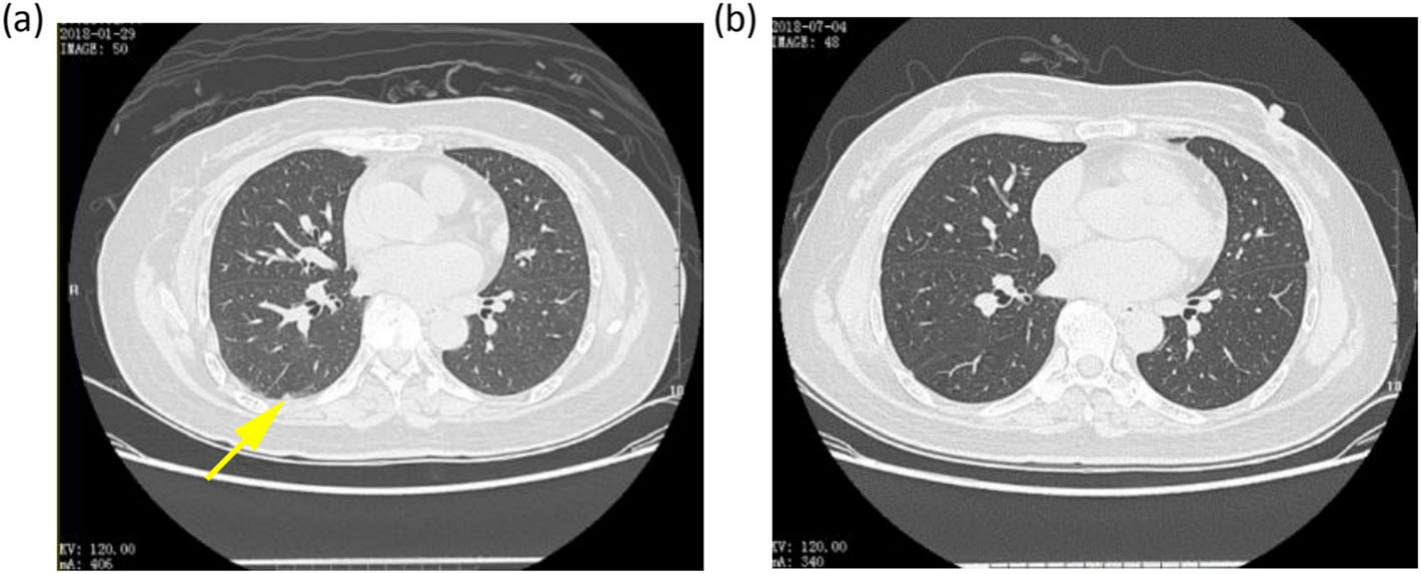
Disappearance of suspected metastatic subpleural nodule at the lower lobe of the right lung in the pulmonary window. **a** Before treatment. **b** After 6 months of apatinib

After taking apatinib for 1 week, the patient found that the tumor in the right lower abdominal wall had begun to shrink and the bleeding had stopped, scabbed and fallen from the wounds in the middle and lower abdomen. During this period, the patient changed the apatinib dosage to 500 mg orally once daily, but she developed dizziness, headache, nausea, vomiting and increased blood pressure (161/103 mmHg). Considering that the patient had no previous history of headache or hypertension, she was treated with antiemetics and drugs to control her blood pressure, and continued to receive the dose of apatinib 250 mg once daily for maintenance. The patient was given apatinib for 8 weeks (Fig. 1b). Follow-up CT scans showed that the multiple subcutaneous masses in the abdominal wall (mainly the anterior and inferior abdominal wall) were significantly smaller and fewer in number than before, and the subpleural nodule at the lower lobe of the right lung had disappeared. The efficacy was assessed as partial response (PR) and the original dose of apatinib was maintained. The CT scan performed after taking apatinib for 5 months (Figs. 3 and 4) indicated that the abdominal lesions continued to shrink and the number of lesions continued to decrease, but the patient stopped taking apatinib 6 months later due to personal reasons, and adopted self-care adjustment. Through a telephone follow-up, we were informed that the patient had no disease progression and a good quality of life. The patient has had a progression-free survival of 19 months.

## Discussion and conclusions

AS is a very rare malignant vascular tumor, accounting for about 1% of the total number of sarcomas. The incidence of AS is generally related to exposure to chemical substances, radiation, chronic lymphedema and trauma. Positive CD31 staining of IHC is the one of the most reliable diagnostic markers, indicating that 80% of AS express both D2–40 and CD31 [6]. Secondary AS are mostly associated with MYC gene amplification [7–9]. In this case, the patient developed abdominal wall AS 8 years after radio-chemotherapy for cervical carcinoma, with CD31 staining positive in IHC and MYC amplification in WES, which indicated for the diagnosis of secondary AS.

AS may be characterized by the up-regulation of vascular-specific receptor tyrosine kinases, including TIE1, VEGF-R2, TEK, and VEGF-R1, and the down-regulation of VEGF ligand expression (VEGF-A and VEGF-B) [10]. On this basis, several scholars have tried to use anti-VEGF targeted drugs, including sorafenib, sunitinib and apatinib [1, 11–13] to treat AS. Apatinib, targeted primarily at VEGF-R2, was approved by the China Food and Drug Administration (CFDA) in 2014 for the third and fourth-line treatment of advanced gastric cancer or adenocarcinomas of the esophagogastric junction [14]. Data from small clinical trials also show that apatinib has a certain efficacy on stage IV sarcomas (ORR 20.0% and DCR 80.0%) [15]. Adverse reactions of apatinib include hypertension, proteinuria, hand-foot syndrome and so on [16–21]. In addition, the use of apatinib for the treatment of AS associated with local bleeding has not been reported, perhaps due to the potential risk of bleeding associated with its use.

In this case, the patient received the dose of apatinib 250 mg once daily, and the lesions stopped bleeding with impressive results. The subsequent WES report indicated that VEGFA, VEGF-R2(KDR), VEGF-R1(FLT1), VEGF-R3(FLT4), TIE, TEK (TIE2) and VEGF-A were all negative, as well as BRAF/HRAS gene co-mutation. In addition to VEGF-R2, apatinib may also inhibit Ret, c-kit, c-SRC and so on [22, 23]. To our knowledge, no reports have that apatinib inhibits BRAF/HRAS mutation.

We report a rare case of a radiation-induced abdominal wall AS patient who had a dramatic response to low-dose apatinib. The WES report indicated consistent BRAF/HRAS gene mutation and MYC amplification. It remains unclear whether this patient’s sensitivity to low-dose apatinib therapy is related to the result of mutations or amplification, and this point requires further study.

## Data Availability

The dataset supporting the conclusions of this article is owned by the First Affiliated Hospital of Guangzhou University of Chinese Medicine but could be made available on request. Personal information will not be provided to ensure anonymity of the patient.

## Abbreviations

AS: angiosarcoma
CT: computed tomography
IHC: immunohistochemistry
RT: radiotherapy
WES: whole exome sequencing
